# Laparoscopic-Assisted ERCP in Gastric Bypass Patients—No Stones Left Unturned: A Single Center Retrospective Cohort Study

**DOI:** 10.1007/s11695-024-07268-4

**Published:** 2024-06-05

**Authors:** B. D. Petersen, C. Nørregaard, R. Krøijer, A. Floyd, M. Ploug

**Affiliations:** 1 Department of Surgical Gastroenterology, Hospital South West Jutland, Region of Southern Denmark, Finsensgade 35, Skolebakken 141, 6705 Esbjerg, Denmark; 2 Department of Surgery, Odense University Hospital, J.B. Winsløws Vej 4, 5000 Odense, Denmark; 3 Department of Regional Health Research, University of Southern Denmark, Odense, Denmark

**Keywords:** Laparoscopic-assisted ERCP, Choledocholithiasis, Gastric bypass, Retrospective study

## Abstract

**Purpose:**

The long-term need for biliary duct intervention following Roux-en-Y gastric bypass surgery (RYGB) is uncertain. We investigated the rate of laparoscopic assisted retrograde cholangiopancreatography (LAERCP) following RYGB. Also, the pre-LAERCP diagnostic workup together with the true rate of choledocholithiasis in patients with or without prior cholecystectomy was investigated.

**Materials and Methods:**

Retrospective cohort study of RYGB and LAERCP performed at the Hospital South West Jutland, University Hospital of Southern Denmark, from 1 January 2013 to 31 May 2022.

**Results:**

One percent of patients (*n* = 13) with a history of RYGB (*n* = 1363) underwent LAERCP at our facility during a median follow-up of 60.6 months. The stone extraction rate was 66.7% in patients with in situ gallbladder and 12.5% in patients with prior cholecystectomy. Cannulation of the common bile duct was achieved in 96.7% of cases. Postoperative complications were observed in 22.6% of the cases.

**Conclusion:**

Approximately 1% of RYGB patients needed LAERCP during a median follow-up of 5 years. In patients with a history of cholecystectomy, the LAERCP rate of stone extraction was very low (12.5%).

**Graphical Abstract:**

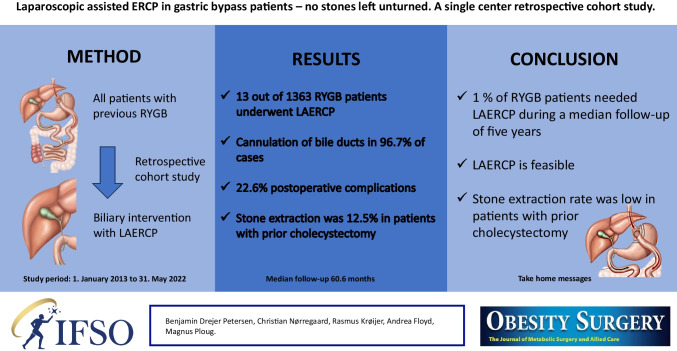

## Introduction

Gallstone disease and gallstone formation are well-known complications after bariatric surgery, and in the first years after bariatric surgery, the risk of cholecystectomy is increased [1]. Roux-en-Y gastric bypass (RYGB) [2] is among the most common procedures in the world [2, 3]. However, following RYGB access to the bile duct with conventional endoscopic retrograde cholangiopancreatography (ERCP) is no longer technically feasible due to the altered intestinal anatomy. As a result, in cases of complicated gallstone diseases necessitating clearance of the bile duct, more invasive procedures are required. The most common procedure in the Nordic countries is the laparoscopic-assisted ERCP (LAERCP). LAERCP is facilitated via laparoscopic intraperitoneal access mostly by insertion of a laparoscopic port into the excluded stomach allowing a standard duodenoscope to be advanced to the papilla of Vateri [4]. The need for such laparoscopy-assisted procedure to access the bile ducts if needed has since been used as an argument against RYGB when comparing it to other bariatric procedures, such as the gastric sleeve, where the regular route to the papilla still is possible. However, while the increase in gallstone formation following RYGB is well described, no estimates are provided for the long-term risk of LAERCP in patients after RYGB.

Outside the setting of bariatric surgery, studies report that 12% of the patients show evidence of common bile duct stones at the time of cholecystectomy [5], while the risk of de novo or retained common bile duct stones following cholecystectomy appears much lower [6]. Whether the risk of common bile duct stones in patients with or without their gallbladder differs in patients with RYGB is not clear. This paucity of knowledge could lead to unnecessary interventions in searching for and treating choledocholithiasis in patients with a low probability of stones. The published studies on LAERCP often present data on technical parameters, such as access to the papilla and cannulation of bile ducts, as well as on adverse events [4, 7]. Other clinically relevant endpoints such as stone extraction, the need for repeat intervention, and the resolution of symptoms are not always reported [7, 8]. LAERCP poses similar complication rates in terms of the endoscopic procedure as ERCP [4, 9]. Complications following ERCP range from mild to life-threatening and include pancreatitis (3–10%), infection (4%), and post-sphincterotomy bleeding (2%) [10].

The primary outcome of this study was to investigate the proportion of patients requiring LAERCP following RYGB. As secondary outcomes, we explored the indications for LAERCP, pre-LAERCP work-up, and stone extraction in patients with or without prior cholecystectomy. Finally, we examined the safety and feasibility of LAERCP in patients with a history of gastric bypass.

## Materials and Methods

### Study Population

In this retrospective cohort study, we included patients with a history of RYGB surgery or LAERCP from the Hospital South West Jutland, University Hospital of Southern Denmark (HSWJ). Patients were included from 1 March 2013 to 31 May 2022, and follow-up ended on 1 April 2023. The Hospital South West Jutland, University Hospital of Southern Denmark is the only center performing post-bariatric LAERCP in that region of Southern Denmark. At our institution, bile duct intervention in patients with a history of RYGB is exclusively performed using the LAERCP procedure. The reporting follows the STROBE guidelines [11].

### Measures and Data Extraction

Residents in Denmark are assigned a unique identification number at birth or upon first entry into the country, the CPR number [12]. This number is used for all healthcare contacts. Based on this number, we searched the administrative systems at our hospital, to identify patients for inclusion. The search was based on the Danish version of the International Classification of Disease classification system (ICD-10) [13] and on the Nordic Medico-Statistical Committee Classification of Surgical Procedures [14].

Relevant patients were captured by combining procedural codes covering endoscopic or transduodenal procedures (KUJK*/KJKE*) on the bile duct together with the registration of the RYGB procedure code (KJDF10/KJDF11) or with the registration of the diagnostic code that the patient is living with a RYGB (DZ980c). We chose a relatively broad spectrum of procedural codes as no specific procedure code for LAERCP exists.

The combination of codes used to identify the study cohort were as follows:

- KJDF1* + KUJK*/KJKE*, where the endoscopic procedure code was registered at a later date than the RYBG procedure code
  and/or
- DZ980c + KUJK*/KJKE* where the endoscopic procedure codes were registered at the same time or later than the registration of DZ980c.

Based on the CPR number, the identified patients were cross-linked to the hospital’s electronic medical records. By manual examination, it was confirmed that the identified procedure codes did in fact cover LAERCP procedures. In addition, the following baseline characteristics were registered: age, sex, height, weight, laboratory results, ASA score, smoking status, indication for LAERCP, radiological investigations prior to LAERCP, access to the papilla, therapeutic success, cannulation of the pancreatic duct, precut sphincterotomy, stone extraction, sphincterotomy, length of hospital stay following LAERCP, repeat LAERCP within 6 months after index procedure, previous or perioperative cholecystectomy, intraoperative events, postoperative complications, and mortality within 90 days after LAERCP. Complications were classified according to the Clavien–Dindo classification system [15].

For patients referred to our institution for LAERCP after RYGB was performed elsewhere, we used July 1st of the respective year when the exact date of the RYGB procedure was unavailable.

### Outcome and Definitions

The primary outcome, the proportion of patients who undergo LAERCP following RYGB, was determined in the subgroup of patients where the RYGB procedure was performed at our institution. The secondary outcomes, examining indications for LAERCP, pre-LAERCP work-up, and stone clearance between patients with or without prior cholecystectomy, were investigated using the entire patient cohort including patients with a history of RYGB performed elsewhere.

Prior cholecystectomy was defined as earlier than 7 days before LAERCP and not within the same hospital admission. Cleared stones from the bile duct were defined to include only those where the extraction of stones was explicitly mentioned in the operative charts of the medical journal. Sludge or gravel was therefore not included.

The safety and feasibility of LAERCP were addressed by examining intraoperative events (both in relation to the laparoscopic and endoscopic part), overall 30-day morbidity, and therapeutic success. Therapeutic success was defined as cannulation of the common bile duct. In case of multiple events in the same patient, the most severe intraoperative event and postoperative complication following the Clavien–Dindo Classification were registered.

### Statistical Analysis

Baseline characteristics were presented using medians for continuous data and by proportions for categorical data. A 95% confidence interval (CI) of proportion was calculated for the primary outcome. Differences between the patient groups with and without prior cholecystectomies regarding stone extraction were analyzed using Fisher’s exact test. A *p*-value < 0.05 was considered statistically significant. No explicit measures were taken to analyze for missing data on emigration or death following the RYGB procedure.

### Approvals

Ethics approval, patient consent, and clinical trial registration are not applicable for retrospective registry-based research according to Danish legislation. The use of patient file data was approved by the Region of Southern Denmark (record-ID 22/60279).

### Operative Approach

The overall technique was relatively similar among the four involved surgeons. Port placement included a 10 mm port near the umbilicus and two 5 mm ports in the left and right upper quadrants. Following a laparoscopic overview and, if necessary, cholecystectomy, the LAERCP procedure was initialized. In patients where same-session cholecystectomy was performed, a guidewire was inserted via the cystic stump to the duodenum enabling the rendezvous technique. A small gastrotomy was created on the anterior surface of the excluded stomach in which a 15 mm trocar was introduced (most often with the port placement being in the upper part of the abdomen in the left midclavicular line). Afterwards, the biliary limb was identified and clamped with a band or with an intestinal grasper to obstruct the lumen to minimize the need for CO_2_ insufflation. The duodenoscope was inserted through the 15 mm trocar into the excluded stomach and the ERCP procedure could be continued. After the ERCP procedure, the duodenoscope and the 15 mm trocar in the excluded stomach were removed. The gastrotomy was closed in two layers using a continuous running suture technique with absorbable sutures.

**Table:** 

Tips and recommendations for a successful laparoscopy-assisted ERCP:
• The qualifications to perform both the ERCP and the bariatric/surgical procedure are preferably available in the same person. This will also lower the administrative burden of co-scheduling two high-qualification medical professionals. • Distal clamping of the biliary limb for better insufflation during endoscopy. • Percutaneous trocar placement directly into the excluded stomach without the use of stay sutures. • Closure of the gastrotomy with a simple two-layer running suture. No use of purse string suturing around the trocar and no use of sutures to adhere the excluded stomach to the anterior abdominal wall.

## Results

**Fig. 1:**
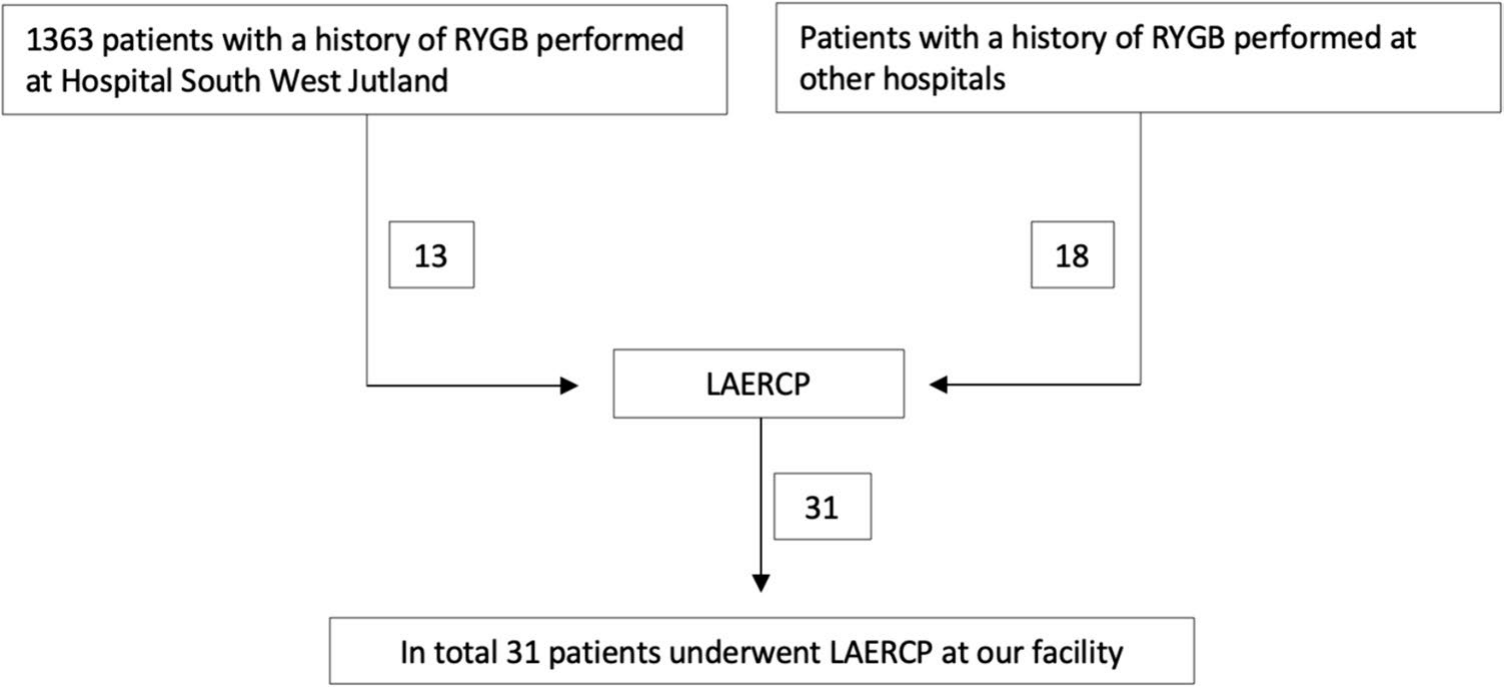
Overview

A total of 1363 patients underwent RYGB at our institution in the study period. Subsequently, 13 patients underwent a LAERCP (0.95%, CI 0.90–1.00%). Median follow-up was 60.6 months (range 10.2–119.2). The median time from RYGB to LAERCP was 56.6 months (range 6.4–185.4). Eighteen individuals with a history of RYGB (performed outside of our facility) underwent LAERCP at our facility (Fig. 1). Overall, four repeat LAERCPs were done within 6 months due to choledochal stent removal placed during the initial LAERCP.

Patient demographics including data on the indication for LAERCP and pre-LAERCP work-up are presented in Table 1.

**Table 1:** Demographics, indications, and pre-operative radiological examinations at the time of LAERCP (*n* = 31)

Median age (range)	49.3	(27.9–62.8)
Median ASA score (range)	2	(1–3)
Female, ***N*** (%)	26	(84%)
Smoking, ***N*** (%)	2	(6.5%)
Median BMI, kg/m^2^ (range)	28	(18.8–51.1)
Median time since cholecystectomy, months (range)	50.8	(1.4–185.4)
Radiological investigation prior to LAERCP		
Ultrasound, *N* (%)	21	(67.7%)
CT, *N* (%)	17	(54.8%)
MRCP, *N* (%)	17	(54.8%)
Indication for LAERCP		
Choledocholithiasis, *N* (%)	22	(70.9%)
Acute calculous cholangitis, *N*	2/22	
Acute biliary pancreatitis, *N*	1/22	
Biliary dyskinesia, *N* (%)	6	(19.3%)
Bile duct injury, *N* (%)	2	(6.5%)
Malignant obstruction, *N* (%)	1	(3.2%)

*ASA*American Society of Anesthesiologists, *BMI*body mass index, *RYGB*Roux−en−Y gastric bypass, *LAERCP*laparoscopic-assisted endoscopic retrograde cholangiopancreatography, *CT*computed tomography, *MRCP*Magnetic resonance cholangiopancreatography

Transabdominal ultrasound was utilized to evaluate the bile ducts in 67.7% of cases. CT and MRCP were both used in 54.8% of cases. In patients with a history of cholecystectomy, MRCP was utilized prior to LAERCP in 87.5% (14/16) of patients compared to 20% (3/15) in patients with their gall bladder in situ. Among patients with a history of cholecystectomy, MRCP was suspicious for choledocholithiasis in four cases. Choledocholithiasis was the main indication for LAERCP in 62.5% (10/16) of patients with prior cholecystectomy and in 85% (12/15) with gallbladder in situ.

### Cholecystectomy and Choledocholithiasis

A total, 51.6% (16/31) had a history of cholecystectomy prior to the LAERCP, while 45% (14/31) had cholecystectomy performed in the same session as the LAERCP. Stone extraction from the bile ducts was more common in patients with gall bladder in situ than in those without (67.7% vs. 12.5%, *p* = 0.003). The difference in the stone extraction rate persisted between the two groups when only examining those where the indication was choledocholithiasis (75% vs. 20%, *p* = 0.03). Of the four cases where MRCP was suspicious of stones in patients with a history of cholecystectomy, one case of choledocholithiasis was confirmed at LAERCP.

### Safety and Feasibility (Tables 2 and 3)

**Table 2:** Summary of operative data and events (*n* = 31)

LAERCP		
Access to the papilla, *N* (%)	31	(100%)
Cannulation of common bile duct, *N* (%)	30	(96.8%)
Precut prior to cannulation, *N* (%)	6	(19.3%)
Sphincterotomy, *N* (%)	27	(87.1%)
Stone extraction, *N* (%)	12	(38.7%)
Unintentional cannulation of ductus pancreaticus, *N* (%)		
Yes	7	(22.6%)
Unknown	12	(38.7%)
No	12	(38.7%)
Perioperative cholecystectomy, *N* (%)	14	(45.1%)
Intraoperative events, *N* (%)		
Gallbladder perforation	5	(35.7%)
Bile duct injury	2	(14.3%)
Conversion to open surgery	None	
Gastric remnant perforation	None	
Major bleeding	None	

**Table 3:** Postoperative data and complications within 90 days (*n* = 31)

Median length of stay after LAERCP, days (range)	2	(0–15)
Repeat LAERCP within 6 months, *N*	4	
Median postoperative P-amylase levels, U/L (range)^1^	48	(4–1810)
Postoperative complications, *N* (%)		
Pancreatitis	3	(9.7%)
Perforations	3	(9.7%)
Hospital readmission	1	(3.3%)
Bleeding, abscess, surgical site infection	None	
Clavien–Dindo classification, *N* (%)		
Grade I–II	4	(12.9%)
Grade III–V	3	(9.7%)
Mortality within 90 days, no (%)	1*	(3.3%)

*LAERCP*laparoscopic assisted endoscopic retrograde cholangiopancreatography

1)Highest measurement in the postoperative period. In three patients no data were available

*Due to disseminated malignant disease and not as a complication of LAERCP

Therapeutic success was achieved in 30 of 31 cases (96.8%). Seven intraoperative events were noted, two of which were bile duct injuries. All events were related to cholecystectomies.

Seven postoperative complications were noted (22.6%). Pancreatitis occurred in three cases, one of which was severe. In another three cases, repeat laparoscopy was needed. One patient had bile leakage from a sutured common hepatic duct; one patient had reactive bile peritonitis related to iatrogenic gall bladder perforation during the same session cholecystectomy; and the last patient was diagnosed with an inflamed sigmoid colon at repeat laparoscopy.

Lastly, one readmission occurred due to abdominal pain, requiring only analgesic treatment. Four complications were classified as CD grade I–II and three complications as CD grade IIIb.

## Discussion

During a 5-year median follow-up of roughly 1400 patients operated with RYGB at our institution, 0.95% (CI 0.90–1.00%) of the patients underwent a subsequent LAERCP. The technical success rate was high, and the need for repeat interventions was low. In patients with prior cholecystectomy, the stone extraction rate was markedly lower when compared to those with their gall bladder in situ.

Limited research exists on the overall need for bile duct interventions following RYGB. The rates between 0.9 and 1.2% are reported on the need for intervention due to choledocholithiasis alone [16–18], yet our study also included patients where LAERCP was done on other indications, including the alleviation of biliary dyskinesia among others. We found that 0.95% of RYGB patients needed intervention during the 5-year follow-up. Considering that we find a similar rate of LAERCP as studies with longer follow up and more narrow criteria for recording LAERCP, it suggests that our estimate is conservative and that the rate would increase had another 5 years been added to the study period.

Choledocholithiasis was the indication for LAERCP in 85% of patients with their gallbladder in situ and 62.5% in patients with a history of cholecystectomy. Stone extraction during LAERCP was observed in only 12.5% of patients with a history of cholecystectomy and in 66.7% of patients with an intact gallbladder or those undergoing same session cholecystectomy. Choledocholithiasis following cholecystectomy is considered infrequent, and the findings in this study suggest that this is also the case in RYGB patients. In patients with a history of prior cholecystectomy, the relatively low rate of stone extraction at LAERCP was surprising considering that 62.5% of these patients were treated on suspicion of stone disease.

Routine administration of Ursodeoxycholic acid following RYGB is not practiced at our institution, and no patients were treated with Ursodeoxycholic acid at the time of LAERCP.

Overall therapeutic success (defined as cannulation of the common bile duct) was achieved in 96.8% of procedures, which is in line with both international and national data [4, 7, 19]. Precut was needed in 19.3% prior to cannulation, which is a considerably higher rate when compared to standard ERCP [20].

Postoperative complications were found in 22.6% of cases, most of them being minor. Three patients (9.7%) developed pancreatitis, which is higher than the 1.4–4.9% reported in systematic reviews [4, 21]. One possible explanation is the observed rate of unintentional cannulation of ductus pancreaticus occurring in 22.6% of cases. Together with the high rate of precut needed, this presumably illustrates the difficulties in positioning the endoscope. Three complications were classified as CD IIIb, because of the need for repeat laparoscopy. In two of these, the complications were attributed to the perioperative cholecystectomy and not the LAERCP. The reported rates of complications in the published literature range from 12.8 to 35.5% [4, 7, 21, 22], figures that seem to be in line with the findings in our study. Given the complication rate of around 20% and that choledocholithiasis was only found in 12.5% of LAERCP procedures performed in patients without a gallbladder, we recommend that thorough diagnostic measures should be carried out before opting for LAERCP. Furthermore, only a quarter of MRCP-diagnosed stones in patients with prior cholecystectomy were confirmed at LAERCP. This suggests that other diagnostic tools could be relevant to investigate prior to LAERCP.

Limitations in the present study should be acknowledged. We did not have access to data in out-patient facilities; consequently, bias due to omission of less severe complications, such as surgical site infection, pneumonia, or minor intraabdominal infections is possible and therefore causing us to underestimate the true complication rate. The retrospective manner of data collection is a limitation as well. We rely on chart review, where data can be missing or might be subject to interpretation. We did not analyze in general for missing data, i.e., patients leaving the Region of Southern Denmark after RYGB or death. Consequently, the rates might be conservative, although they seem in line with previously published studies. Lastly, the relatively strict definition of “stone” in our study might cause us to underestimate the rate of stone extraction.

## Conclusion

Approximately 1% of RYGB patients needed LAERCP during a median follow-up of 5 years. In patients with a history of cholecystectomy, the LAERCP rate of stone extraction was very low (12.5%). This surprising finding warrants further research into better diagnostic tools for diagnosing choledocholithiasis in RYGB patients.
